# Uninephrectomy and sodium‐glucose cotransporter 2 inhibitor administration delay the onset of hyperglycemia

**DOI:** 10.14814/phy2.70121

**Published:** 2024-11-10

**Authors:** Yuri Sakai Ishizaki, Masao Kikuchi, Koichi Kaikita, Shouichi Fujimoto

**Affiliations:** ^1^ Division of Cardiovascular Medicine and Nephrology, Department of Internal Medicine, Faculty of Medicine University of Miyazaki Miyazaki Japan

**Keywords:** chronic kidney disease, diabetes, glucose homeostasis, glucose reabsorption, sodium‐glucose cotransporters

## Abstract

The kidneys are essential for glucose homeostasis, as they perform gluconeogenesis, utilize glucose, and reabsorb glucose. Reabsorption is performed by SGLT2, which is responsible for about 90%. However, little is known about how renal glucose handling is altered in patients with chronic kidney disease (CKD). SGLT2 inhibitors have demonstrated efficacy in suppressing CKD progression in clinical trials, but their mechanisms are not fully understood. Therefore, this study aimed to evaluate how each uninephrectomy (UNx) and SGLT2 inhibitor affects blood glucose concentrations and SGLTs dynamics in rats with type 2 diabetes mellitus. Male rats were divided into four treatment groups: sham + placebo, sham + dapagliflozin, UNx + placebo, and UNx + dapagliflozin. There were few group differences in food intake or body weight, but blood glucose concentrations continued to rise in the sham + placebo, whereas this rise was delayed for several weeks in the UNx + placebo, and largely suppressed by dapagliflozin. SGLT2 mRNA expression was significantly lower in the UNx group, but SGLT1 mRNA expression did not significantly differ. Dapagliflozin did not alter SGLT1 or SGLT2 mRNA expression. In animal models of diabetes, renal glucose reabsorption appears likely to be a major contributor to the development of hyperglycemia.

## 1 INTRODUCTION

The kidneys play essential roles in three processes involved in glucose homeostasis: gluconeogenesis, glucose utilization, and glucose reabsorption (Alsahli & Gerich, 2017). Glucose‐6‐phosphatase, a key enzyme for gluconeogenesis, is only found in the liver and kidneys in humans and other animals, with renal gluconeogenesis first being reported by Benoy et al. in 1937 (Benoy & Elliott, 1937). The kidneys usually produce about 25% of the total glucose generated through gluconeogenesis; however, the rate of renal gluconeogenesis can be increased to match that of the liver during prolonged fasting. Renal gluconeogenesis occurs mostly in the cortex, where the activity of glucose‐6‐phosphatase, fructose‐1,6‐bisphosphatase, and phosphoenolpyruvate carboxykinase is high. The kidneys also consume glucose produced in the body, with glycolysis occurring in the renal medulla. The partial pressure of oxygen is low in this area of the kidneys, and glucose is therefore metabolized anaerobically (Neuhofer & Beck, 2005). The most important role of the kidneys in glucose homeostasis is in glucose reabsorption. Almost all the glucose filtered by the glomerulus (approximately 180 g per day) is reabsorbed by sodium‐glucose cotransporters (SGLTs) in the proximal tubules, with SGLT2 being responsible for about 90% of this volume.

While the kidneys clearly play an important role in glucose homeostasis, how this function is altered by chronic kidney disease (CKD) has not been well studied. Many clinicians have noted improved glycemic control in diabetic patients as renal dysfunction has progressed (Ahmad et al., 2019), with this at least in part being because of the concomitant decrease in renal gluconeogenesis. In patients with type 2 diabetes, glucose released from both the kidneys and liver contributes to elevated fasting blood glucose concentrations (Meyer et al., 1998). However, the increase in the rate of gluconeogenesis in the kidneys is approximately 3‐fold, relative to non‐diabetic patients, whereas there is only a 1.3‐fold increase in the rate of gluconeogenesis in the liver. In addition, postprandial renal gluconeogenesis is increased in patients with type 2 diabetes. The decreased clearance of insulin and other glucose‐lowering drugs as renal dysfunction progresses is also thought to play a role in the improvement in glycemic control in these patients. Patients with non‐diabetic CKD have also been reported to have decreased insulin sensitivity and clearance compared with healthy controls (de Boer et al., 2016), and living kidney donors also have decreased insulin sensitivity after nephrectomy (Tanriover et al., 2021). With regard to the renal reabsorption of glucose, this is approximately 3.5‐fold higher during fasting and 2‐fold higher after meals in diabetic patients than in healthy controls (Meyer et al., 1998). However, it is unclear how this changes as renal dysfunction progresses, with some reports showing an increase in the expression of SGLT2 mRNA and protein (Rahmoune et al., 2005; Tabatabai et al., 2009; Wang et al., 2017), while others show a decrease in the expression (Solini et al., 2017; Srinivasan Sridhar et al., 2019).

Most of the glucose reabsorption occurring in the kidneys is regulated by SGLT2 in the early proximal tubules. Although SGLT2 inhibitors have emerged as glucose‐lowering agents through their ability to induce glycosuria, several large trials, including DAPA‐CKD (Heerspink et al., 2020) and EMPA‐KIDNEY (The EMPA‐KIDNEY Collaborative Group et al., 2023), have shown that SGLT2 inhibitors also slow the progression of CKD in patients with and without diabetes mellitus. SGLT2 inhibitors appear to protect the kidneys by decreasing intraglomerular pressure via tubuloglomerular feedback (Khunti, 2021), as well as by improving glucose metabolism, reducing oxidative stress, and mitigating the mTORC1 pathway (Schaub et al., 2023). However, these mechanisms have not yet been fully elucidated.

In this study, we examined the effects of uninephrectomy (UNx) and SGLT2 inhibitor administration on the dynamics of blood glucose concentrations and SGLTs mRNA expression in diabetic model rats. This aimed to improve the understanding of renal glucose handling, and to help reduce the onset and progression of type 2 diabetes mellitus and diabetic nephropathy.

## 2 MATERIALS AND METHODS

This study was approved by the University of Miyazaki Animal Research Committee (approval number: 2023–505). The authors confirm that all the experiments were performed according to approved guidelines.

### 2.1 Rat model of diabetic nephropathy

Spontaneously Diabetic Torii (SDT) fatty rats, a model strain of obese type 2 diabetes mellitus with early‐onset hyperglycemia, were purchased from CLEA Japan Inc., Tokyo, Japan. This strain of rats was developed through the introduction of a leptin receptor mutation (Leprfa/Leprfa).

### 2.2 UNx and dapagliflozin administration study

Five‐week‐old male SDT fatty rats were housed in single cages; 12 h light/dark cycles and fed a normal Charles River Formula (CRF‐1) diet (Oriental Yeast Co., Ltd. Tokyo, Japan) and water ad libitum until 6 weeks of age, and were then randomly assigned to four treatment groups: (i) sham + placebo (*n* = 6), (ii) sham + dapagliflozin (*n* = 6), (iii) UNx + placebo (*n* = 6), and (iv) UNx + dapagliflozin (*n* = 6). The UNx and sham operations were performed at 6 weeks of age using inhalation anesthesia with isoflurane and the dapagliflozin‐treated groups had dapagliflozin (Namiki Shoji Co., Ltd.) administered in water at a dose of 1.5 mg/kg/day while measuring the amount of drinking water ad libitum. The placebo groups continued to have water provided ad libitum, and the CRF‐1 diet was provided ad libitum to all treatment groups throughout the experiment. The rats were euthanized by inhalation with isoflurane and exsanguination at 12 weeks of age, and their kidneys were sampled for real‐time quantitative polymerase chain reaction (qRT‐PCR) analysis. The sham + dapagliflozin group was additionally kept until 30 weeks of age (*n* = 3).

### 2.3 Measurement of biological parameters

The rats were housed in metabolic cages overnight (for an average period of 17 h) once a week from 6 to 12 weeks of age to measure urine volume, food intake, and water consumption. Body weight and blood pressure (measured using tail‐cuff plethysmography) were also determined weekly. Serum creatinine and blood glucose concentrations in blood samples collected from the tail vein were measured weekly using analysis kits (creatinine kit: StatSensor Xpress®, Nova Biomedical Cooperation, USA; blood glucose kit: FreeStyle Precision, Abbott, USA).

### 2.4 Continuous glucose monitoring

To examine fluctuations in blood glucose concentrations more closely, glucose concentrations were continuously monitored using a novel flash glucose monitoring system (FreeStyle Libre, Abbott, UK). The devices were inserted into the rats' abdominal cavities 4 days before the UNx procedure. The surgery was performed painlessly using inhalation anesthesia with isoflurane and analgesics with subcutaneous buprenorphine.

### 2.5 Kidney tissue processing and qRT‐PCR assay

Kidney tissue samples were harvested when the rats were sacrificed at 12 weeks of age. Diced aliquots (60 mg) of kidney cortex were suspended in RLT/β‐mercaptoethanol buffer (RNeasey kit, Qiagen, Germantown, MD, USA) and then frozen at −80°C. RNA from the kidney cortex was purified using an RNeasy mini kit (catalog no. 74106, Qiagen, Germantown, MD, USA), followed by reverse transcription into complementary DNA (cDNA) using a high‐capacity cDNA reverse transcription kit (catalog no. 4368814, Applied Biosystems, Foster City, CA, USA). qRT‐PCR was performed using a LightCycler 96® system (Roche Molecular System, Inc.). The qRT‐PCR data were used to calculate the relative expressions of the target genes, SGLT2 and SGLT1, using glyceraldehyde‐3‐phosphate dehydrogenase (GAPDH) for comparison, and phosphoenolpyruvate carboxykinase (Pck1) and pyruvate kinase (Pklr), using β‐actin (Actb) for comparison. The TaqMan probes (Applied Biosystems) used were: rat SGLT2 (catalog no. Rn00574917_m1), rat SGLT1 (catalog no. Rn01640634_m1), rat GAPDH (catalog no. Rn01775763_m1), rat Pck1 (catalog no. Rn01529014_m1), rat Pklr (catalog no. Rn01455286_m1), and rat Actb (catalog no. Rn00667869_m1).

### 2.6 Western blot (WB) assay

SGLT2 protein expression was evaluated using western blot analysis. Briefly, 100 mg samples of kidney tissue from the renal cortex were homogenized in 600 μL of RIPA buffer (catalog no. 16648834, Nacalai Tesque, Inc., Japan) at 12,000x g for 30 min at 4°C. The protein concentration was tested using a PierceTM bicinchoninic acid (BCA) protein assay kit (catalog no. A55865, Thermo Fisher Scientific, USA), and samples containing 50 μg of protein were separated using 10% SDS‐PAGE and transferred to polyvinylidene difluoride membranes (catalog no. 1620177, Bio‐Rad Laboratories, Inc., USA). The membranes were blocked in 5% non‐fat dry milk and incubated with the relevant antibodies. The primary antibodies used for detection were SGLT2 (catalog no. ab37296, Abcam), SGLT1 (catalog no. ab14686, Abcam), and β‐actin (catalog no. ab8227, Abcam), with mouse anti‐rabbit IgG‐HRP (catalog no. sc‐2357, Santa Cruz Biotechnology) being used as the secondary antibody. After incubation with the secondary antibody, the protein bands were visualized using the enhanced chemiluminescence kit (catalog no. 1705060, Bio‐Rad Laboratories, Inc., USA). The bands were analyzed using a Fusion FX imaging system (Vilber, France). β‐actin was used to normalize the results of each sample.

### 2.7 Statistical analysis

**FIGURE 1:**
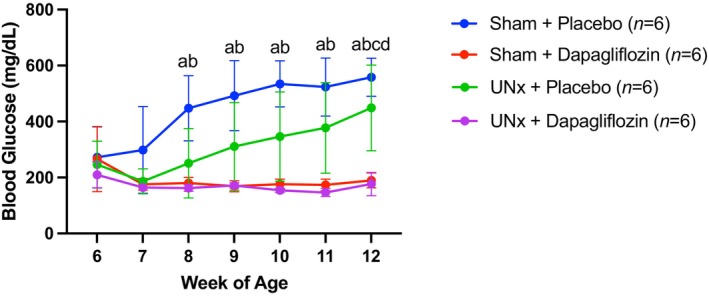
The effects of uninephrectomy (UNx) and/or dapagliflozin administration on blood glucose concentrations in SDT fatty rats from 6 to 12 weeks of age. Error bars indicate the standard deviation, and statistically significant differences (*p* < 0.05) between every two groups are shown by letters (a: Sham + placebo vs. sham + dapagliflozin, b: Sham + placebo vs. UNx + dapagliflozin, c: Sham + dapagliflozin vs. UNx + placebo, d: UNx + placebo vs. UNx + dapagliflozin) using mixed model, with Bonferroni correction.

GraphPad PRISM software, version 10.0 (GraphPad Softwere, Inc., USA) and JMP Pro (JMP Statistical Discovery LLC, USA) were used for statistical analysis. All data except Figure 4 are presented as the mean ± standard deviation. All statistical data were tested for normality, and since normality was present, the means of variables in two or more independent groups were compared using the unpaired *t*‐test and ANOVA with Bonferroni correction in all figures except Figure 4. The data were analyzed using the Mann–Whitney tests for the placebo group in Figure 4 because normality was absent. In addition, the differences in blood glucose levels in each group in Figure 1 were statistically analyzed using a mixed model. *p* values <0.05 were considered statistically significant. Data from six rats in each group were analyzed.

## 3 RESULTS

### 3.1 Changes in blood glucose concentration

**FIGURE 2:**
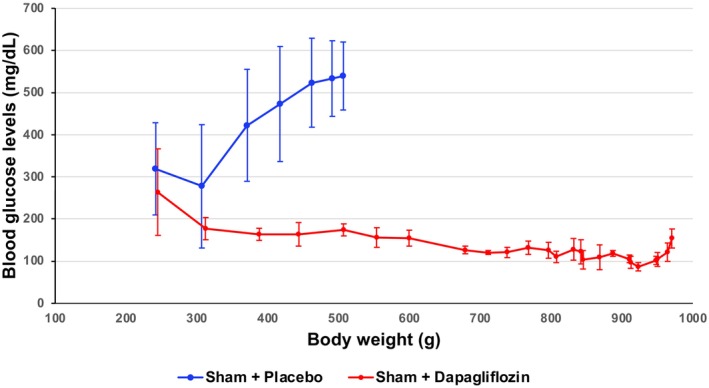
The effects of dapagliflozin administration on blood glucose concentrations in the sham group of SDT fatty rats from 6 to 30 weeks of age. Error bars indicate the standard deviation. The sham + placebo and the sham + dapagliflozin groups were observed from 6 to 12 weeks and from 6 to 30 weeks, respectively.

Figure 1 shows that blood glucose concentrations continued to rise from 8 weeks of age in the sham + placebo group, whereas this rise was delayed for 4 weeks in the UNx + placebo group. There was a significant difference in the change in blood glucose levels over time between the UNx + placebo group and the sham + placebo group (*p* = 0.033). Dapagliflozin administration suppressed this rise in blood glucose concentration in both UNx rats, which only had a single kidney, and sham rats, which still had both kidneys. In addition, the increase in blood glucose levels was suppressed in the sham + dapagliflozin group until 30 weeks of age, even when the body weight reached 1 kg (Figure 2).

### 3.2 Changes in body weight, feed and water consumption, and urinary output

**FIGURE 3:**
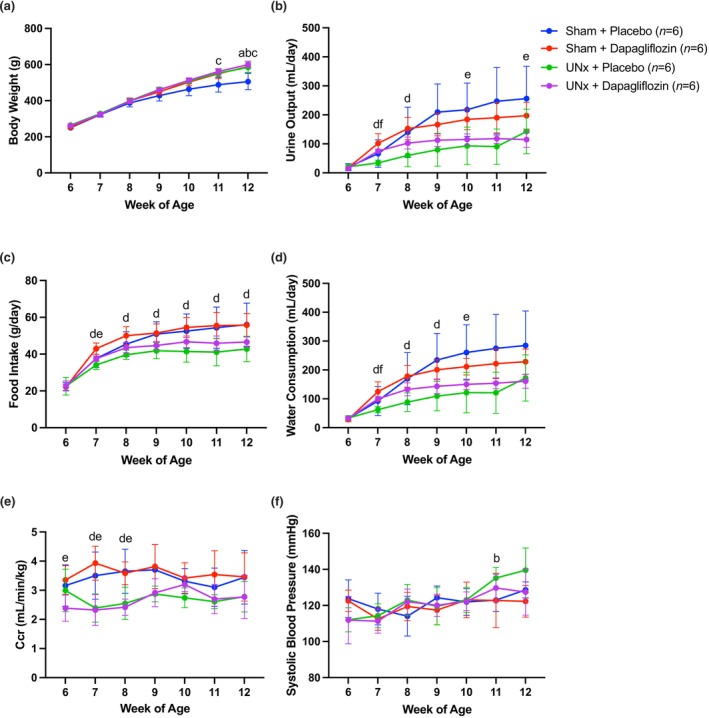
The effects of uninephrectomy (UNx) and/or dapagliflozin administration on body weight (a), 24‐h urinary output (b), 24‐h food intake (c), 24‐h water consumption (d), creatinine clearance (e), and systolic blood pressure (f) in SDT fatty rats from 6 to 12 weeks of age. Error bars indicate the standard deviation, and statistically significant differences (*p* < 0.05) between every two groups are shown by letters (a: Sham + placebo vs. sham + dapagliflozin, b: Sham + placebo vs. UNx + placebo, c: Sham + placebo vs. UNx + dapagliflozin, d: Sham + dapagliflozin vs. UNx + placebo, e: Sham + dapagliflozin vs. UNx + dapagliflozin, f: UNx + placebo vs. UNx + dapagliflozin) using two‐way ANOVA, with Bonferroni correction. Ccr: Creatinine clearance.

Rats in the sham + placebo group were prone to increased urinary output volumes secondary to hyperglycemia and had decreased body weights after 11 weeks of age, whereas rats in the other treatment groups continued to gain weight throughout the experiment (Figure 3a,b). Feed consumption in the dapagliflozin treatment groups increased (Figure 3c), but blood glucose concentrations were suppressed. The dapagliflozin treatment group drank more water than placebo before 10 weeks of age and tended to increase urinary output from 7 weeks of age (Figure 3d). The UNx group showed that creatinine clearance decreased after uninephrectomy, but did not differ from the sham group after weeks (Figure 3e). The only significant difference in blood pressure was found between the UNx + placebo group and the sham + dapagliflozin group at 11 weeks of age (Figure 3f).

### 3.3 Continuous glucose monitoring

**FIGURE 4:**
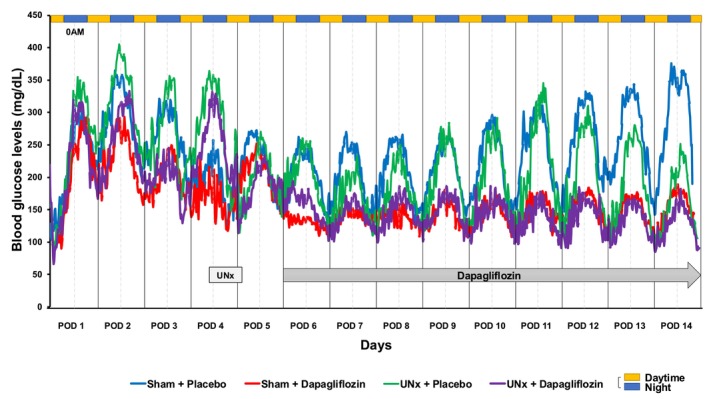
The effects of uninephrectomy (UNx) and/or dapagliflozin administration on continuously monitored blood glucose concentrations in SDT fatty rats over a 14‐day period. POD: Postoperative day, UNx: Uninephrectomy, AM: Ante meridiem.

Blood glucose concentrations were continuously monitored for 2 weeks, beginning 4 days before the UNx or Sham surgery. Peak blood glucose concentrations were usually observed late at night, as expected in a nocturnal species. Flush glucose monitoring showed that blood glucose levels were lower in the UNx group than in the sham group, but postprandial glucose concentrations decreased by 20% (*p* < 0.0001), whereas fasting glucose concentrations decreased by only 4% (*p* = 0.026) (Figure 4). Severe hypoglycemia was not observed in either of the two dapagliflozin treatment groups.

### 3.4 SGLT2 mRNA expression

**FIGURE 5:**
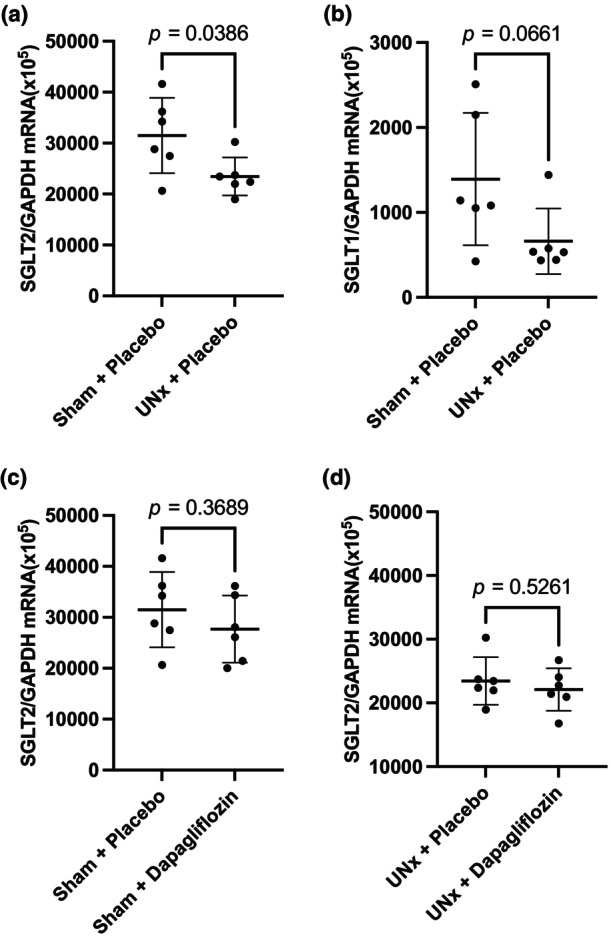
The effects of uninephrectomy (UNx) and/or dapagliflozin administration on sodium‐glucose cotransporter (SGLT) mRNA expression in the kidney cortex tissue of SDT fatty rats. (a) SGLT2/GAPDH mRNA ratios in the sham and UNx treatment groups. (b) SGLT1/GAPDH mRNA ratios in the sham and UNx treatment groups. (c) SGLT2/GAPDH mRNA ratios in rats treated with either dapagliflozin or the placebo in the sham group. (d) SGLT2/GAPDH mRNA ratios in rats treated with either dapagliflozin or the placebo in the UNx group. Results are indicated as mean ± standard deviation, and the data were analyzed using unpaired *t*‐tests.

We determined the SGLT2 and SGLT1 mRNA expression using qRT‐PCR to investigate the reason for the delayed onset of hyperglycemia in the UNx + placebo treatment group. As can be seen in Figure 5a, SGLT2 mRNA expression in the renal cortex was significantly (26%) lower in the UNx group than in the sham group (*p* = 0.0386), whereas SGLT1 mRNA expression did not significantly differ between these two groups (Figure 5b). Dapagliflozin administration did not significantly alter SGLT2 mRNA expression in either the sham or UNx groups (Figure 5c,d).

### 3.5 SGLT protein abundance

**FIGURE 6:**
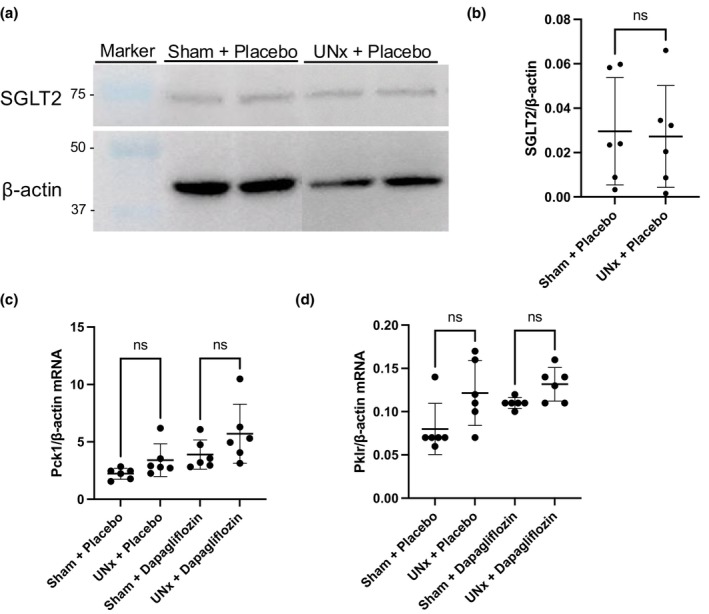
The effects of uninephrectomy (UNx) and/or dapagliflozin administration on sodium‐glucose cotransporter (SGLT) protein, phosphoenolpyruvate carboxykinase (Pck1), and pyruvate kinase (Pklr) expression in the kidney tissue of SDT fatty rats. (a) Western blot analysis using antibodies against SGLT2 and β‐actin in the sham and UNx groups, (b) SGLT2/β‐actin protein abundance in the sham and UNx groups (mean ± standard deviation). (c) Pck1/β‐actin mRNA ratios in the four treatment groups (mean ± standard deviation). (d) Pklr/β‐actin mRNA ratios in the four treatment groups (mean ± standard deviation). Data were evaluated using one‐way ANOVA, with Bonferroni correction. ns: Not significant.

SGLT2 and SGLT1 protein abundance were examined by western blotting to further probe the relationship between UNx and SGLTs in diabetic rats (Figure 6a). No SGLT1 bands were detected (data not sown). As shown in Figure 6b, there was no significant difference in SGLT2 abundance between the sham and UNx groups.

### 3.6 Evaluation of gluconeogenesis and glycolysis

To further elucidate the cause of the delayed rise in blood glucose in the UNx + placebo group, the mRNA expression of Pck1 and Pklr, which are relatively well‐expressed in the kidney, were examined using qRT‐PCR. Pck1 and Pklr mRNA expression were used to assess the status of gluconeogenesis and glycolysis processes, respectively. Results showed no differences between the sham and UNx treatment groups (Figure 6c,d).

## 4 DISCUSSION

We performed UNx on type 2 diabetes mellitus model rats to study glucose homeostasis under CKD conditions, and showed that the onset of hyperglycemia was delayed in the UNx rats, relative to the rats that had undergone a sham operation. A possible mechanism for this delay was an observed decrease in SGLT2 mRNA expression. When dapagliflozin, an SGLT2 inhibitor, was administered to the UNx and sham surgery treatment groups, the onset of hyperglycemia was largely suppressed in both groups, but no significant change in SGLT2 mRNA expression was observed.

Hypoglycemia is a common complication in patients with CKD, and is particularly prevalent in patients who also have diabetes (Moen et al., 2009). Meta‐analysis has shown that the incidence of hypoglycemia is significantly higher in patients with CKD than in patients without CKD (relative risk = 1.89, 95% confidence interval = 1.86–1.92, *p* < 0.0001) (IA et al., 2020).

We investigated the mechanism of the inhibition of hyperglycemia observed in this study. The kidneys are involved in glucose homeostasis through their participation in the processes of gluconeogenesis, glucose utilization, and glucose reabsorption. The mRNA expression of Pck1, which is responsible for gluconeogenesis, did not significantly differ between the UNx and sham surgery treatment groups. The mRNA expression of Pklr, which is involved in the glycolytic system, also did not significantly differ between the UNx group and the sham surgery group. This suggests that the delayed onset of hyperglycemia induced by UNx is not related to changes in glyconeogenesis or the glycolytic system, and is therefore likely predominantly influenced by renal glucose reabsorption.

The effects of UNx have been well studied with respect to contralateral compensatory hypertrophy, but little has been done with respect to glucose homeostasis. Also, much remains to be elucidated regarding the regulation of SGLT2 mRNA expression. In animals (Kato et al., 2005) and living renal transplant donors (Tanriover et al., 2021), it has been reported that insulin concentrations are increased after UNx compared to preoperative concentrations, but blood glucose concentrations are similar to preoperative concentrations, that is, insulin resistance. To adapt under insulin resistance, it may regulate glucose balance in the kidney via a decrease in SGLT2 mRNA expression. Indeed, renal receptors for glucagon (GCGR), which raises blood glucose concentrations, have also been reported to be decreased in CKD (Bonner et al., 2015; Wang et al., 2024), allowing for similar considerations. Another mechanism is that renal GCGRs are mainly located in the thick part of the nephron, but also in the same proximal tubules as SGLT2. Magnetic resonance imaging studies report that the remaining kidney hypertrophies by about 24% within a few days after UNx (Song et al., 2014), suggesting that stress on the renal tubules from the rapid changes in glomerular filtration rate may have affected the expression of both receptors at the same site. In the glomerulus, increased cellular stress in apparently healthy human allografts has been shown using cell‐specific transcriptional changes in podocytes (Menon et al., 2022). Although UNx is considered safe for healthy subjects, such as living renal donors, recent cohort studies have reported that these subjects are at higher risk of end‐stage kidney disease in the long term (Mjøen et al., 2014; Muzaale et al., 2014). The results of this study may contribute to our understanding of glucose homeostasis in living kidney donors.

The effect of dapagliflozin on the onset of hyperglycemia in diabetic rats observed in this study was significant, with the increase in blood glucose concentrations during the observation period being almost completely suppressed in the dapagliflozin treatment groups. This effect was observed over a long period of time, even in the presence of severe obesity. The effect of dapagliflozin on new‐onset type 2 diabetes has been previously assessed in a pooled analysis of patient‐level data from the DAPA‐CKD and DAPA‐HF trials (Heerspink et al., 2020; McMurray et al., 2019; Mori et al., 2022; Rossing et al., 2022). Over a median follow‐up period of 21.2 months, 126 (6.3%) of 2008 patients in the placebo group and 85 (4.3%) of 1995 patients in the dapagliflozin group developed type 2 diabetes (hazard ratio = 0.67, 95% confidence interval = 0.51–0.88, *p* = 0.0040). Although this study only included patients enrolled in the DAPA‐CKD and DAPA‐HF trials, and thus only included patients with CKD and/or heart failure, dapagliflozin is expected to also be effective in preventing new‐onset type 2 diabetes in patients with a background of insulin resistance. In addition, no severe hypoglycemia was observed in the UNx group treated with dapagliflozin during continuous glucose monitoring in our study, suggesting that dapagliflozin can be safely administered to patients with CKD.

One limitation of this study was that the observation period, during which mRNA expression changes were measured and the efficacy of dapagliflozin was evaluated, corresponded to only the early stages of diabetes onset. The SDT fatty rats were also in the early stages of CKD, with little or no change in renal function. Further studies with longer observation periods are therefore needed to clarify the results of this study. Secondly, the study used a minimum number of rats for the experiment from the perspective of animal welfare, so there are limitations to the statistical power of the study. The third limitation is that only a limited number of the enzymes involved in gluconeogenesis and glycolysis were investigated, and not all of them were investigated and discussed.

## 5 PERSPECTIVES AND SIGNIFICANCE

In summary, UNx delayed the onset of hyperglycemia in diabetic rats via the down‐regulation of SGLT2 mRNA expression. The SGLT2 inhibitor, dapagliflozin, largely suppressed the onset of hyperglycemia but did not alter SGLT2 mRNA expression. These data suggest that renal glucose reabsorption appears likely to play a major role in the development of hyperglycemia in animal models of diabetes, even under CKD conditions.

## AUTHOR CONTRIBUTIONS

Y Ishizaki and M Kikuchi designed the research. Y Ishizaki performed the experiments. Y Ishizaki and M Kikuchi analyzed and interpreted the data. Y Ishizaki prepared figures and drafted the first manuscript. Y Ishizaki, M Kikuchi, K Kaikita, and S Fujimoto edited and revised the manuscript. All authors approved the final version of the manuscript.

## FUNDING INFORMATION

This work was supported by a grant for Clinical Research from Miyazaki University Hospital (no number).

## CONFLICT OF INTEREST STATEMENT

The authors declare no conflicts of interest.

## DISCLAIMERS

The content is the responsibility of the authors alone and does not necessarily represent the official views or policies of the International University of Health and Welfare, nor does mention of trade names, commercial products, or organizations imply endorsement by the government of Japan.

## Data Availability

Full data are available on request.

## References

[phy270121-bib-0001] Ahmad, I. , Zelnick, L. R. , Batacchi, Z. , Robinson, N. , Dighe, A. , Manski‐Nankervis, J. E. , Furler, J. , O'Neal, D. N. , Little, R. , Trence, D. , Hirsch, I. B. , Bansal, N. , & de Boer, I. H. (2019). Hypoglycemia in people with type 2 diabetes and CKD. Clinical Journal of the American Society of Nephrology, 14(6), 844–853. 10.2215/CJN.11650918 30996047 PMC6556736

[phy270121-bib-0002] Alsahli, M. , & Gerich, J. E. (2017). Renal glucose metabolism in normal physiological conditions and in diabetes. Diabetes Research and Clinical Practice, 133, 1–9. 10.1016/j.diabres.2017.07.033 28866383

[phy270121-bib-0003] Benoy, M. P. , & Elliott, K. A. (1937). The metabolism of lactic and pyruvic acids in normal and tumour tissues: Synthesis of carbohydrate. The Biochemical Journal, 31(8), 1268–1275. 10.1042/bj0311268 16746453 PMC1267071

[phy270121-bib-0004] Bonner, C. , Kerr‐Conte, J. , Gmyr, V. , Queniat, G. , Moerman, E. , Thévenet, J. , Beaucamps, C. , Delalleau, N. , Popescu, I. , Malaisse, W. J. , Sener, A. , Deprez, B. , Abderrahmani, A. , Staels, B. , & Pattou, F. (2015). Inhibition of the glucose transporter SGLT2 with dapagliflozin in pancreatic alpha cells triggers glucagon secretion. Nature Medicine, 21(5), 512–517. 10.1038/nm.3828 25894829

[phy270121-bib-0005] de Boer, I. H. , Zelnick, L. , Afkarian, M. , Ayers, E. , Curtin, L. , Himmelfarb, J. , Ikizler, T. A. , Kahn, S. E. , Kestenbaum, B. , & Utzschneider, K. (2016). Impaired glucose and insulin homeostasis in moderate‐severe CKD. Journal of the American Society of Nephrology, 27(9), 2861–2871. 10.1681/ASN.2015070756 26823551 PMC5004653

[phy270121-bib-0006] Heerspink, H. J. L. , Stefánsson, B. V. , Correa‐Rotter, R. , Chertow, G. M. , Greene, T. , Hou, F. F. , Mann, J. F. E. , McMurray, J. J. V. , Lindberg, M. , Rossing, P. , Sjostrom, C. D. , Toto, R. D. , Langkilde, A. M. , & Wheeler, D. C. (2020). Dapagliflozin in patients with chronic kidney disease. The New England Journal of Medicine, 383(15), 1436–1446. 10.1056/NEJMoa2024816 32970396

[phy270121-bib-0007] ALEissa, M. S. , AlGhofaili, I. A. , Alotaibe, H. F. , Yaslam, M. T. , AlMujil, M. S. , Arnous, M. M. , & Al Dalbhi, S. K. (2020). Incidence and risk factors associated with hypoglycemia among patients with chronic kidney disease: A systematic review. Journal of Family & Community Medicine, 27(3), 157–162. 10.4103/jfcm.JFCM_304_19 33354145 PMC7745784

[phy270121-bib-0008] Kato, Y. , Ohno, Y. , Hayashi, M. , Suzawa, T. , Shibagaki, K. , Sasaki, T. , & Saruta, T. (2005). Divergent effects of unilateral and subtotal nephrectomy on insulin sensitivity in rats. Renal Failure, 27(4), 451–457. 10.1081/JDI-65346 16060135

[phy270121-bib-0009] Khunti, K. (2021). SGLT2 inhibitors in people with and without T2DM. Nature Reviews Endocrinology, 17(2), 75–76. 10.1038/s41574-020-00453-2 33293703

[phy270121-bib-0010] McMurray, J. J. V. , Solomon, S. D. , Inzucchi, S. E. , Køber, L. , Kosiborod, M. N. , Martinez, F. A. , Ponikowski, P. , Sabatine, M. S. , Anand, I. S. , Bělohlávek, J. , Böhm, M. , Chiang, C. E. , Chopra, V. K. , de Boer, R. A. , Desai, A. S. , Diez, M. , Drozdz, J. , Dukát, A. , Ge, J. , … DAPA‐HF Trial Committees and Investigators . (2019). Dapagliflozin in patients with heart failure and reduced ejection fraction. The New England Journal of Medicine, 381(21), 1995–2008. 10.1056/NEJMoa1911303 31535829

[phy270121-bib-0011] Menon, R. , Otto, E. A. , Berthier, C. C. , Nair, V. , Farkash, E. A. , Hodgin, J. B. , Yang, Y. , Luo, J. , Woodside, K. J. , Zamani, H. , Norman, S. P. , Wiggins, R. C. , Kretzler, M. , & Naik, A. S. (2022). Glomerular endothelial cell‐podocyte stresses and crosstalk in structurally normal kidney transplants. Kidney International, 101(4), 779–792. 10.1016/j.kint.2021.11.031 34952098 PMC9067613

[phy270121-bib-0012] Meyer, C. , Stumvoll, M. , Nadkarni, V. , Dostou, J. , Mitrakou, A. , & Gerich, J. (1998). Abnormal renal and hepatic glucose metabolism in type 2 diabetes mellitus. The Journal of Clinical Investigation, 102(3), 619–624. 10.1172/JCI2415 9691098 PMC508922

[phy270121-bib-0013] Mjøen, G. , Hallan, S. , Hartmann, A. , Foss, A. , Midtvedt, K. , Øyen, O. , Reisæter, A. , Pfeffer, P. , Jenssen, T. , Leivestad, T. , Line, P. D. , Øvrehus, M. , Dale, D. O. , Pihlstrøm, H. , Holme, I. , Dekker, F. W. , & Holdaas, H. (2014). Long‐term risks for kidney donors. Kidney International, 86(1), 162–167. 10.1038/ki.2013.460 24284516

[phy270121-bib-0014] Moen, M. F. , Zhan, M. , Hsu, V. D. , Walker, L. D. , Einhorn, L. M. , Seliger, S. L. , & Fink, J. C. (2009). Frequency of hypoglycemia and its significance in chronic kidney disease. Clinical Journal of the American Society of Nephrology, 4(6), 1121–1127. 10.2215/CJN.00800209 19423569 PMC2689888

[phy270121-bib-0015] Mori, Y. , Duru, O. K. , Tuttle, K. R. , Fukuma, S. , Taura, D. , Harada, N. , Inagaki, N. , & Inoue, K. (2022). Sodium‐glucose cotransporter 2 inhibitors and new‐onset type 2 diabetes in adults with prediabetes: Systematic review and meta‐analysis of randomized controlled trials. The Journal of Clinical Endocrinology and Metabolism, 108(1), 221–231. 10.1210/clinem/dgac591 36217306

[phy270121-bib-0016] Muzaale, A. D. , Massie, A. B. , Wang, M. C. , Montgomery, R. A. , McBride, M. A. , Wainright, J. L. , & Segev, D. L. (2014). Risk of end‐stage renal disease following live kidney donation. JAMA, 311(6), 579–586. 10.1001/jama.2013.285141 24519297 PMC4411956

[phy270121-bib-0017] Neuhofer, W. , & Beck, F. X. (2005). Cell survival in the hostile environment of the renal medulla. Annual Review of Physiology, 67, 531–555. 10.1146/annurev.physiol.67.031103.154456 15709969

[phy270121-bib-0018] Rahmoune, H. , Thompson, P. W. , Ward, J. M. , Smith, C. D. , Hong, G. , & Brown, J. (2005). Glucose transporters in human renal proximal tubular cells isolated from the urine of patients with non‐insulin‐dependent diabetes. Diabetes, 54(12), 3427–3434. 10.2337/diabetes.54.12.3427 16306358

[phy270121-bib-0019] Rossing, P. , Inzucchi, S. E. , Vart, P. , Jongs, N. , Docherty, K. F. , Jhund, P. S. , Køber, L. , Kosiborod, M. N. , Martinez, F. A. , Ponikowski, P. , Sabatine, M. S. , Solomon, S. D. , DeMets, D. L. , Bengtsson, O. , Lindberg, M. , Langkilde, A. M. , Sjöstrand, M. , Stefansson, B. V. , Karlsson, C. , … DAPA‐CKD and DAPA‐HF Trial Committees and Investigators . (2022). Dapagliflozin and new‐onset type 2 diabetes in patients with chronic kidney disease or heart failure: Pooled analysis of the DAPA‐CKD and DAPA‐HF trials. The Lancet Diabetes and Endocrinology, 10(1), 24–34. 10.1016/S2213-8587(21)00295-3 34856173

[phy270121-bib-0020] Schaub, J. A. , AlAkwaa, F. M. , McCown, P. J. , Naik, A. S. , Nair, V. , Eddy, S. , Menon, R. , Otto, E. A. , Demeke, D. , Hartman, J. , Fermin, D. , O'Connor, C. L. , Subramanian, L. , Bitzer, M. , Harned, R. , Ladd, P. , Pyle, L. , Pennathur, S. , Inoki, K. , … Bjornstad, P. (2023). SGLT2 inhibitors mitigate kidney tubular metabolic and mTORC1 perturbations in youth‐onset type 2 diabetes. The Journal of Clinical Investigation, 133(5), e164486. 10.1172/JCI164486 36637914 PMC9974101

[phy270121-bib-0021] Solini, A. , Rossi, C. , Mazzanti, C. M. , Proietti, A. , Koepsell, H. , & Ferrannini, E. (2017). Sodium‐glucose co‐transporter (SGLT)2 and SGLT1 renal expression in patients with type 2 diabetes. Diabetes, Obesity & Metabolism, 19(9), 1289–1294. 10.1111/dom.12970 28419670

[phy270121-bib-0022] Song, T. , Fu, L. , Huang, Z. , He, S. , Zhao, R. , Lin, T. , & Wei, Q. (2014). Change in renal parenchymal volume in living kidney transplant donors. International Urology and Nephrology, 46(4), 743–747. 10.1007/s11255-013-0592-y 24178754

[phy270121-bib-0023] Srinivasan Sridhar, V. , Ambinathan, J. P. N. , Kretzler, M. , Pyle, L. L. , Bjornstad, P. , Eddy, S. , Cherney, D. Z. , & Reich, H. N. (2019). Renal SGLT mRNA expression in human health and disease: A study in two cohorts. American Journal of Physiology. Renal Physiology, 317(5), F1224–F1230. 10.1152/ajprenal.00370.2019 31545924 PMC6879935

[phy270121-bib-0024] Tabatabai, N. M. , Sharma, M. , Blumenthal, S. S. , & Petering, D. H. (2009). Enhanced expressions of sodium‐glucose cotransporters in the kidneys of diabetic Zucker rats. Diabetes Research and Clinical Practice, 83(1), e27–e30. 10.1016/j.diabres.2008.11.003 19095325 PMC2652566

[phy270121-bib-0025] Tanriover, B. , Lingvay, I. , Ahmed, F. , Sandikci, B. , Mohan, S. , Cremers, S. , Karmally, W. , Mohan, P. , Newhouse, J. , Ragunathan, S. , AbdulRahim, N. , Ariyamuthu, V. K. , Ratner, L. E. , & Cohen, D. J. (2021). Insulin sensitivity after living donor nephrectomy. Transplantation Proceedings, 53(6), 1858–1864. 10.1016/j.transproceed.2021.06.007 34246476 PMC8384711

[phy270121-bib-0026] The EMPA‐KIDNEY Collaborative Group , Herrington, W. G. , Staplin, N. , Wanner, C. , Green, J. B. , Hauske, S. J. , Emberson, J. R. , Preiss, D. , Judge, P. , Mayne, K. J. , Ng, S. Y. A. , Sammons, E. , Zhu, D. , Hill, M. , Stevens, W. , Wallendszus, K. , Brenner, S. , Cheung, A. K. , Liu, Z. H. , … Haynes, R. (2023). Empagliflozin in patients with chronic kidney disease. The New England Journal of Medicine, 388(2), 117–127. 10.1056/NEJMoa2204233 36331190 PMC7614055

[phy270121-bib-0027] Wang, M. Y. , Zhang, Z. , Zhao, S. , Onodera, T. , Sun, X. N. , Zhu, Q. , Li, C. , Li, N. , Chen, S. , Paredes, M. , Gautron, L. , Charron, M. J. , Marciano, D. K. , Gordillo, R. , Drucker, D. J. , & Scherer, P. E. (2024). Downregulation of the kidney glucagon receptor, essential for renal function and systemic homeostasis, contributes to chronic kidney disease. Cell Metabolism, 36(3), 575–597. 10.1016/j.cmet.2023.12.024 38237602 PMC10932880

[phy270121-bib-0028] Wang, X. X. , Levi, J. , Luo, Y. , Myakala, K. , Herman‐Edelstein, M. , Qiu, L. , Wang, D. , Peng, Y. , Grenz, A. , Lucia, S. , Dobrinskikh, E. , D'Agati, V. D. , Koepsell, H. , Kopp, J. B. , Rosenberg, A. Z. , & Levi, M. (2017). SGLT2 protein expression is increased in human diabetic nephropathy: SGLT2 protein inhibition decreases renal lipid accumulation, inflammation, and the development of nephropathy in diabetic mice. The Journal of Biological Chemistry, 292(13), 5335–5348. 10.1074/jbc.M117.779520 28196866 PMC5392679

